# Reassessing BMI-based access to joint replacement surgery

**DOI:** 10.1371/journal.pmed.1005003

**Published:** 2026-03-24

**Authors:** Jonathan P. Evans, Joanna McLaughlin, Jonathan T. Evans

**Affiliations:** 1 University of Exeter Medical School, Exeter, United Kingdom; 2 Royal Devon University Healthcare NHS Foundation Trust, Exeter, United Kingdom; 3 University of Bristol, Bristol, United Kingdom; 4 NHS England, South West Regional Team, United Kingdom

## Abstract

In this Perspective, Jonathan Evans and colleagues discuss why restricting access to joint replacement surgery based on BMI alone is not supported by evidence, and highlight how such restrictions risk exacerbating stigma, inequity and avoidable harm to those who would benefit from surgery.

## Teaser

Joint replacement surgery transforms lives for patients with higher body mass index (BMI). Strong evidence shows safe outcomes and meaningful benefit, yet access remains restricted by BMI alone, an approach that risks stigma, inequity, and avoidable harm.

Joint replacement surgery is one of the most successful operations in contemporary medicine, providing substantial pain relief and improved mobility to millions of patients worldwide. However, access to hip and knee replacement is increasingly restricted for people with higher body mass index (BMI), commonly due to concerns about surgical risk, complications and cost. These concerns are not unfounded; higher BMI is associated with increased relative risk of adverse outcomes, particularly periprosthetic joint infection [1]. However, contemporary analyses from national joint registries, comprising millions of procedures, may assist in providing a more reliable assessment of both risk and benefit.

International guidance reflects this nuance. In the United States and Canada, consensus recommendations conditionally support proceeding with total joint arthroplasty across body mass index (BMI) categories, including severe obesity, without mandatory weight reduction, explicitly noting that the evidence supporting rigid BMI thresholds is indirect and of low quality [2,3]. These guidelines emphasize the absence of convincing evidence that postponing surgery to prioritize weight loss improves outcomes, and highlights the need for shared decision-making and transparency about risk.

In the UK, National Institute for Health and Care Excellence (NICE) guidance is explicit that people with osteoarthritis should not be excluded from referral for joint replacement on the basis of being overweight or obese alone [4]. Despite this, the UK is the only country with widespread formal BMI-based restrictions on access to surgery. In 2025, one-third of commissioning organizations in England applied policies contradicting NICE guidance, while many others required evidence of weight loss attempts [5].

Qualitative and population-level studies suggest such policies are driven primarily by short-term financial pressures rather than clinical benefit [6]. Regions implementing strict BMI thresholds experience reduced surgical activity, longer waits and widening socioeconomic disparities, without evidence of improved outcomes. Delayed patients often present with worse symptoms and higher BMI, suggesting that such policies may worsen health rather than improve it [7].

Internationally, the prevalence of BMI-based restrictions has increased steadily since 2013, with many regions introducing mandatory waiting periods or absolute BMI thresholds before referral [2,8]. Depending on local thresholds, patients may face prolonged delays or be denied referral entirely. Given that more than 60% of adults in high socioeconomic countries are overweight or obese, and that around one in 10 will require hip or knee replacement during their lifetime, these policies affect tens of thousands of people each year, disproportionately impacting more deprived populations where both obesity prevalence and musculoskeletal disease burden are higher [7].

Furthermore, the development of ambulatory (outpatient) joint replacement has driven variable use of BMI thresholds at the institution and surgeon level. Adherence to a BMI < 40 guideline has been shown to reduce perioperative emergency department visits for knee arthroplasty, though the impact on hip arthroplasty outcomes is less clear and weight reduction before surgery may paradoxically increase all-cause readmissions in some cases [9].

## Evidence from National Joint Registries

The UK National Joint Registry (NJR) is the most powerful source of evidence for this question. It contains more than 4 million procedures with near complete capture of NHS and independent sector activity. This scale provides extremely precise estimates across the full spectrum of BMI and avoids the selection bias that limits smaller series. The registry includes linked outcomes for revision, mortality, and validated patient reported outcome measures.

The most comprehensive evidence on BMI comes from the NJR study of almost half a million knee replacements [10]. Patients in every BMI category, including class two (BMI 35–39.9) and class three (BMI ≥ 40) obesity, achieved improvements in the Oxford Knee Score well above clinically important thresholds. Although those with higher BMI entered surgery with worse symptoms, their absolute gains were equivalent to other groups. Differences between BMI groups were below the minimum detectable change. Mortality was not higher in patients with raised BMI and was lower than in normal weight groups. Ten-year revision rates remained well below accepted benchmarks. Comparable findings are seen in hip replacement cohorts [11]. These data confirm that BMI alone should not be used as a sole eligibility criterion.

Although the weight of evidence relates to hip and knee arthroplasty, consistent findings have been reported across other joints in large national cohorts. A multinational registry analysis of shoulder replacement from the United Kingdom and Denmark found no increase in mortality, serious adverse events or revision among patients with higher BMI, with some outcomes worse in underweight patients than in any obesity category [12].

However, these results must be interpreted in context. Patients included in registry analyses have been assessed and deemed suitable for surgery by anesthetic and medical teams. The evidence therefore supports surgery in patients with higher BMI who are otherwise appropriate candidates, rather than justifying blanket exclusion of all individuals above an arbitrary threshold.

## Clinical decision-making and weight stigma as barriers to healthcare

Regardless of formal policies, decisions about surgery are ultimately made within the clinical encounter. Surgeons must balance potential benefits against recognized risks, often under conditions of heightened scrutiny, performance monitoring, and limited system capacity. These pressures can understandably promote risk-averse practice, particularly where outcomes are closely audited.

However, evidence indicates that BMI alone is a poor discriminator of benefit or harm. Professional responsibility, therefore, lies in ensuring that patients are supported to make informed choices based on individual risk profiles, rather than being excluded through implicit or explicit thresholds that do not reflect the available data.

Weight loss is often presented as a prerequisite for surgery, yet durable, clinically meaningful weight loss is difficult to achieve without intensive support. Observational studies suggest that rapid or poorly supported preoperative weight loss may increase complications, including infection, potentially through loss of muscle mass, nutritional deficiency, and reduced physiological reserve [13]. Weight optimization should therefore be supportive rather than mandatory, and not used to justify prolonged delay or denial of surgery.

Moreover, BMI-based restrictions do not exist in isolation; they are part of a wider pattern of weight stigma that affects people living with obesity in multiple areas of healthcare. A recent review highlights that weight stigma operates at structural, interpersonal, and internalized levels and that BMI cutoffs represent a clear structural barrier that disproportionately harms those already facing disadvantage [14]. Many healthcare professionals hold negative attitudes toward people with obesity and patients frequently report feeling judged or dismissed. This can lead to avoidance of healthcare and delayed presentation. The notion that weight is entirely within individual control reinforces blame and obscures the biological and social determinants of obesity. Against this backdrop, BMI-based denial of joint replacement can deepen mistrust and exacerbate suffering.

## Towards fair and evidence-based policy

To move toward fair and evidence-based care, BMI must not be used as a barrier to joint replacement. Decisions should be based on holistic assessment of health and risk, not a single metric that was never intended for individual clinical decision-making. Health optimization support should be offered early and equitably, and not as a mandatory requirement immediately before surgery. Guidance should be implemented consistently across healthcare systems to prevent local variation that harms equity. Ultimately, the goal must be to ensure that patients with advanced arthritis receive timely access to interventions that restore quality of life, rather than being delayed or denied because of unreliable assumptions about BMI.

Joint replacement is a highly effective, life improving operation. The evidence from the large-scale international studies is unequivocal: people with high BMI experience substantial benefit, with safe outcomes and similar improvements in pain and function to those with lower BMI. BMI-based restrictions do not reflect this evidence and instead risk perpetuating inequality, worsening symptoms, and causing avoidable harm. It is time to bring policy into alignment with the data and ensure equitable access to joint replacement for all who need it.

## Abbreviations

BMI: body mass index
NICE: National Institute for Health and Care Excellence
NJR: National Joint Registry
