# Correction: Reassessing BMI-based access to joint replacement surgery

**DOI:** 10.1371/journal.pmed.1005076

**Published:** 2026-04-29

**Authors:** The PLOS Medicine Staff

## Notice of Republication

This article was republished on April 16th, 2026 to remove Figure 1, which was published in error. The publisher apologizes for the errors. Please download this article again to view the correct version.
